# INTERGENERATIONAL DIGITAL SOLIDARITY IN AGING FAMILIES AND ITS ASSOCIATIONS WITH RELATIONAL OUTCOMES

**DOI:** 10.1093/geroni/igac059.2328

**Published:** 2022-12-20

**Authors:** Xiaoyu Fu, Woosang Hwang, Merril Silverstein

**Affiliations:** Syracuse University, Syracuse, New York, United States; Syracuse University, Syracuse, New York, United States; Syracuse University, Syracuse, New York, United States

## Abstract

Since the coronavirus disease outbreak, older adults have been isolated from family, as in-person contact declined, and many turned to digital contact to stay in touch. This form of contact, consisting of texting, email, and social media, is labeled digital solidarity. A key advantage of digital communication over in-person contact is that it requires less investment of time and no geographic proximity. However, it is unclear whether digital solidarity represents a separate dimension of intergenerational solidarity, and whether it compensates for low in-person contact. In this paper, we examined traditional and digital types of intergenerational communication between older parents and adult children, and their associations with older adults’ perceived quality of communication and closeness with children. We used the 2016 wave of the Longitudinal Study of Generations to generate a sample of 580 older parents who reported on relationships with 1,489 adult children. Adopting a three-step latent class approach, we identified four classes of intergenerational communication: all-type contact, no contact, digital contact, and traditional contact. Older adults in both no contact and digital contact classes were less likely to report being emotionally close with their adult children when compared to those in the traditional contact class. No difference in perceived quality of communication was found between contact classes. Our findings indicate that digital solidarity is a distinct dimension of intergenerational solidarity and can compensate for reduced in-person contact with children. Discussion centers on the implications of these results for pandemic times and a replication using recently collected data from 2021-22.

